# Effect of Respiratory Growth on the Metabolite Production and Stress Robustness of *Lactobacillus casei* N87 Cultivated in Cheese Whey Permeate Medium

**DOI:** 10.3389/fmicb.2019.00851

**Published:** 2019-04-24

**Authors:** Annamaria Ricciardi, Teresa Zotta, Rocco Gerardo Ianniello, Floriana Boscaino, Attilio Matera, Eugenio Parente

**Affiliations:** ^1^Scuola di Scienze Agrarie, Forestali, Alimentari e Ambientali, Università degli Studi della Basilicata, Potenza, Italy; ^2^Istituto di Scienze dell’Alimentazione – Consiglio Nazionale delle Ricerche (CNR), Avellino, Italy; ^3^Dipartimento di Scienze, Università degli Studi della Basilicata, Potenza, Italy

**Keywords:** *Lactobacillus casei*, whey permeate, respiration, pyruvate metabolism, diacetyl, stress survival

## Abstract

Cheese whey permeate (WP) is a low-cost feedstock used for the production of biomass and metabolites from several lactic acid bacteria (LAB) strains. In this study, *Lactobacillus casei* N87 was cultivated in an optimized WP medium (WPM) to evaluate the effect of anaerobic and respiratory conditions on the growth performances (kinetics, biomass yield), consumption of sugars (lactose, galactose, glucose) and citrate, metabolite production [organic acids, volatile organic compounds (VOCs)] and stress survival (oxidative, heat, freezing, freeze-drying). The transcription of genes involved in the main pathways for pyruvate conversion was quantified through Real Time-PCR to elucidate the metabolic shifts due to respiratory state. Cultivation in WPM induced a diauxic growth in both anaerobic and respiratory conditions, and *L. casei* N87 effectively consumed the lactose and galactose present in WPM. Genomic information suggested that membrane PTS system and tagatose-6-P pathway mediated the metabolism of lactose and galactose in *L. casei* N87. Respiration did not affect specific growth rate and biomass production, but significantly altered the pyruvate conversion pathways, reducing lactate accumulation and promoting the formation of acetate, acetoin and diacetyl to ensure the redox balance. Ethanol was not produced under either cultivation. Pyruvate oxidase (*pox*), acetate kinase (*ack*), α-acetolactate decarboxylase (*ald*), acetolactate synthase (*als*) and oxaloacetate decarboxylase (*oad*) genes were up-regulated under respiration, while L-lactate dehydrogenase (*ldh*), pyruvate formate lyase (*pfl*), pyruvate carboxylase (*pyc*), and phosphate acetyltransferase (*pta*) were down regulated by oxygen. Transcription analysis was consistent with metabolite production, confirming that POX-ACK and ALS-ALD were the alternative pathways activated under aerobic cultivation. Respiratory growth affected the production of volatile compounds useful for the development of aroma profile in several fermented foods, and promoted the survival of *L. casei* N87 to oxidative stresses and long-term storage. This study confirmed that the respiration-based technology coupled with cultivation on low-cost medium may be effectively exploited to produce competitive and functional starter and/or adjunct cultures. Our results, additionally, provided further information on the activation and regulation of metabolic pathways in homofermentative LAB grown under respiratory promoting conditions.

## Introduction

Cheese whey (CW) is one of the main by-products of dairy industry (Prazeres et al., 2012). CW is recognized as polluting effluent because of high chemical (COD) and biochemical (BOD) oxygen demand levels, and its management and disposal is still a relevant problem in dairy sector, from both economical (large volumes; high waste treatment costs) and environmental (inadequate waste disposal) point of view (Prazeres et al., 2012; Carvalho et al., 2013). Different physicochemical (e.g., protein precipitation, membrane separation) and biological (i.e., microbial conversion of lactose) treatments have been developed and used over time to decontaminate CW, to reduce the organic content and to produce value-added compounds from CW and CW components (Prazeres et al., 2012; Atamer et al., 2013).

CW and/or ultrafiltrated CW (deproteinized whey permeate, WP) have been extensively exploited as alternative low-cost feedstock (lactose source, nutrient supplement) for the production of biomass (high cell-density cultures) and/or lactic acid from several lactose-fermenting lactic acid bacteria (LAB) (Tuli et al., 1985; Aeschlimann and von Stockar, 1989; Audet et al., 1989; Krischke et al., 1991; Parente and Zottola, 1991; Norton et al., 1994; Arasaratnam et al., 1996; Kulozik and Wilde, 1999; Tango and Ghaly, 1999; Fitzpatrick et al., 2001; Schepers et al., 2002; Panesar et al., 2007, 2010; Cui et al., 2012; Prasad et al., 2014; Patel and Parikh, 2016). The composition of whey-based substrates (e.g., supplementation with carbon and nitrogen sources, minerals, vitamins; as LAB have complex nutritional requirements) and the operating conditions (e.g., temperature, pH, inoculum size, cell system, oxygen demand, type of cultivation) were also optimized to boost the efficiency and productivity of fermentation processes (Panesar et al., 2007; Prazeres et al., 2012). Supplemented whey-media have been also used for the production of other organic compounds (acetic acid, propylene glycol; Veeravalli and Mathews, 2018; acetoin, diacetyl, Gutierrez et al., 1996; Nadal et al., 2009; ethanol, Liu et al., 2016b), bacteriocins (e.g., nisin, pediocin, plantaricin; Liao et al., 1993; Bertrand et al., 2001; Guerra et al., 2001; Liu et al., 2005; Enan et al., 2006; Jozala et al., 2011) and exopolysaccharides (Macedo et al., 2002a,b) from different LAB. Supplemented CW and WP, additionally, have been exploited as growth media and/or thermoprotectants to improve the survival of some probiotic lactobacilli to spray-drying treatments (Lavari et al., 2014, 2015; Hugo et al., 2016; Huang S. et al., 2018). More recently, mozzarella CW has been also used for biogas production (Pagliano et al., 2019).

The fitness of LAB in a given set of conditions (e.g., culture media, ecological niches, fermented foods, hosts), however, mainly depends on the type of metabolism. Although LAB are conventionally recognized as anaerobic oxygen-tolerant microorganisms, several authors (see Zotta et al., 2017a for a review) have demonstrated that conditions promoting aerobic respiration (presence of oxygen, supplementation with heme or heme and menaquinone for the activation of a minimal respiratory chain) induce in some LAB the expression of phenotypes with improved physiological and technological traits. The capability to activate a minimal respiratory chain and/or other oxygen-related pathways has been investigated in some homofermentative strains of *Lactococcus lactis*, *Lactobacillus casei*, *L. plantarum*, and *Streptococcus agalactiae* (Yamamoto et al., 2005; Brooijmans et al., 2009; Lechardeur et al., 2011; Zotta et al., 2017a), and more recently in some heterofermentative species (*L. reuteri*, *L. spicheri*, Ianniello et al., 2015; *L. brevis*, *L. fermentum*, *Leuconostoc mesenteroides*, Zotta et al., 2018). These studies have addressed the effect of aerobic respiration on the growth performances and stress robustness of LAB, highlighting the significant differences with cells grown anaerobically, and elucidating some molecular mechanisms involved in the activation and regulation of respirative-mediated pathways.

To date, studies of the potential benefits of respiratory phenotypes have been carried out in synthetic complex (e.g., M17, MRS, Weissella Medium broth) and/or chemically defined media (Zotta et al., 2017a). Recently, Reale et al. (2016b) found that cells of *L. casei* N87 and N2014 grown under respiratory conditions, compared to anaerobically ones, prevented oxidative processes (e.g., free radical accumulation, peroxidation of proteins and lipids) and increased desirable aroma compounds (e.g., acetoin and diacetyl) in Cheddar-type cheeses, suggesting that starter and adjunct cultures produced with respiration-based technology could be of practical relevance in dairy sector.

Moreover, since *L. casei* also includes strains characterized and exploited as probiotics (Hill et al., 2018; Huang C. et al., 2018), the study and the understanding of aerobic and respiratory metabolism may be useful to produce more robust and competitive cultures.

In this study, we used a WP medium (WPM) to cultivate the respiration-competent strain *L. casei* N87 under anaerobic (AN) and respiratory (RS) conditions (oxygen and supplementation with hemin and menaquinone). The effect of cultivation (RS vs. AN) on the growth performances, sugar consumption, metabolite production and stress robustness (i.e., oxidative, heat, freezing, freeze-drying) was evaluated and compared with previous data obtained in synthetic complex media. The use of respiration-technology and WP as low-cost substrate for the production of important aroma compounds (e.g., acetoin, diacetyl) was also investigated. The transcription of genes involved in the main pathways for pyruvate conversion was quantified through Real Time-PCR to elucidate the metabolic shifts due to respiratory cultivation and to define the oxygen-responsive pathways; the consistency between gene expression and metabolite production was also verified.

## Materials and Methods

### Strain and Culture Conditions

*Lactobacillus casei* N87 (Ianniello et al., 2016) was maintained as freeze-dried stock (11% w/v skim milk containing 0.1% w/v ascorbic acid) in the Culture Collection of the Laboratory of Industrial Microbiology, Università degli Studi della Basilicata, and routinely propagated in Weissella Medium Broth, pH 6.8 (WMB; Zotta et al., 2012), for 16 h at 37°C.

### Preparation of Whey Permeate

Whey permeate (WP) was obtained from partially defatted and ultrafiltrated pasta filata cheese whey as described by Lavari et al. (2015). WP containing 38.01 ± 0.25 g/l of lactose and 3.82 ± 0.60 g/l of galactose (measured with enzymatic kits, R-Biopharm AG, Darmstadt, Germany) was stored at -20°C until use.

### Batch Cultivations in Whey Permeate Medium

*L. casei* N87 was cultivated at 37°C in the sterile optimized WP medium (WPM; WP supplemented with 2.5 g/l yeast extract, 2.5 g/l tryptone, 0.1 g/l MgSO_4_⋅7H_2_O, 0.02 g/l MnSO_4_⋅H_2_O, 0.5 ml/l Tween 80; Lavari et al., 2015) under anaerobic (AN, nitrogen 0.1 vol/vol/min) or respiratory (RS, 60% dissolved oxygen, supplementation of WPM with 2.5 μg/ml hemin and 1 μg/ml menaquinone) conditions. Bioreactors (2.5 l working volume; Applikon, Schiedam, the Netherlands) were inoculated (2% v/v) with an overnight (16 h, 37°C) anaerobic WMB pre-culture, washed twice in 20 mM potassium phosphate buffer pH 7.0 (PB7) and standardized to an optical density at 650 nm (OD_650_) of 2.0 (SmartSpec^TM^130 Plus, Bio-Rad Laboratories).

Dissolved oxygen concentration (% dO_2_) was measured using a polarographic electrode (Applisens, Applikon) and was automatically controlled (*ez*Control controller, Applikon; set point 60% = 4.2 mg/l O_2_) by varying air flow and stirrer speed as described in Ianniello et al. (2016). pH was controlled at 6.5 by automatic addition of sterile 4 eq/l NaOH, while foaming was controlled by automatic addition of a sterile 5% (v/v) Antifoam 204 solution. Two biological replicates were carried out for each growth condition.

Samples were aseptically withdrawn for the measurement of OD_650_ until the end of growth. In the first (E1; 5 h) and second exponential (E2; 29 h) growth phases (a diauxic trend was detected of both AN and RS cultivation), the cell dry weight (CDW, g/l) was measured by separating the biomass at 10,000 *× g*, 10 min, 4°C, washing twice in deionized water and drying at 105°C for 24 h. A standard curve correlating OD_650_ values and CDW was obtained by linear regression and used to calculate the biomass production (g/l) during AN and RS growth. The ln-transformed biomass values were used to estimate growth kinetics and parameters with the primary dynamic model of Baranyi and Roberts (1994) using DMFit v.3.5 for Excel (see Baranyi, 2015 for manual instruction; download package at https://www.combase.cc/index.php/en/8-category-en-gb/21-tools). Growth curves were splitted (primary and secondary growth) and, for each cultivation, two independent fit were modeled.

### Chemical and Biochemical Analyses

All analyses were carried out on samples collected in E1 (5 h), S1 (12 h), E2 (29 h), and S2 (42 h) phases. Briefly, the capability to consume oxygen was assessed by re-suspending the cells (standardized to OD_650_ = 1.0) in air-saturated PB7 containing 5.5 mmol/l glucose and 0.002 g/l of resazurin, and measuring the time (h) of resazurin discoloration as indicator of oxygen uptake (Ricciardi et al., 2014). Residual lactose, galactose glucose and citrate, and production of lactate, acetate, ethanol were measured with enzymatic kits (R-Biopharm AG, Darmstadt, Germany). H_2_O_2_ was measured by mixing the cultures supernatants with a mixture containing 4-amino-antipyrine (3 mmol/l), sodium 3,5-dichloro-2 hydroxybenzenesulfonate (10 mmol/l) and peroxidase (0.28 U/ml); the residual amounts of H_2_O_2_ were spectrophotometrically measured at 510 nm (Risse et al., 1992). Sterile WPM was used as control. All analyses were performed on each biological replicate.

### Measurement of Volatile Organic Compounds

Volatile organic compounds (VOCs) were evaluated according to Reale et al. (2016b). VOCs were extracted from sterile WPM (control) and from culture supernatants by using the Solid Phase MicroExtraction (SPME) and analyzed with Gas Chromatography coupled to Mass Spectrometry (SPME/GC-MS). Briefly, a 50/30 μm divinylbenzene/carboxen/polydimethylsiloxane fiber (Supelco, Bellefonte, PA, United States) was introduced into 20 ml headspace vial (5 g of sample/vial) and exposed at 45°C for 30 min. 3-octanol was used as internal standard (5 μl of 100 mg/l standard solution). After extraction, VOCs were desorbed at 230°C for 15 min into the injection port of an Agilent 7890A gas-chromatograph (Agilent Technologies, United States) coupled to an Agilent 5975 quadrupole mass spectrometer, equipped with an Agilent HP-INNOWax capillary GC column (30 m, ID 0.25 mm, 0.25 μm film thickness). Helium was used as carrier gas at a flow rate of 1.5 ml/min. The injection (split/splitless mode, 0.75 mm ID liner) was performed with an oven T °C set to rise from 40 to 230°C, at 5°C/min. Mass spectrometer operated in scan mode over mass range 20–400 amu (2 s/scan), at 70 eV. Identification of volatile compounds was performed by matching the mass spectra against those of reference compounds included in the Wiley v.7 Mass Spectral library (NIST 05). The amount of individual VOCs was expressed as Relative Peak Area (RPA) respect to the internal standard area. Analytical standards 3-hydroxy-2-butanone (range 0–0.5 g/l in water with 0.1% v/v ethanol) and 2,3-butanedione (range 0–0.5 g/l in water with 0.1% v/v ethanol) were used, respectively, to quantify (mg/l) the amounts of acetoin and diacetyl in the culture supernatants and WPM control. VOC profiling in the different growth phases (E1, 5 h; S1, 12 h; E2, 29 h; S2, 42 h) was measured for each biological replicate.

### Radical Scavenging Activity and Survival to Stress Treatments

Radical scavenging activity and stress tolerance were measured on standardized (final OD_650_ = 1.0 in PB7) cell suspensions collected in E1 (5 h), S1 (12 h), E2 (29 h), and S2 (42 h) phases. Hydroxyl-radicals scavenging was evaluated as described by Wang et al. (2009). Briefly, a mixture containing 0.5 ml of 1,10-phenantroline (0.75 mmol/l), 1 ml of 20 mmol/l phosphate buffer pH 7.4 and 0.5 ml of FeSO_4_ (0.75 mmol/l) were added to 0.5 ml of H_2_O_2_ (0.12% v/v) and 0.5 ml of cells. Two control solutions (c1, containing only 1,10-phenantroline, FeSO_4_ and hydrogen peroxide and c2, containing only 1,10-phenantroline and FeSO_4_) were used. Reaction mixtures were incubated a 37°C for 90 min and the content of hydroxyl free radicals was measured at 536 nm. Percentage (%) of scavenging activity was calculated as (%) = (A_s_ -A_c1_)/(A_c2_ -A_c1_) × 100, where A_s_ was the OD_536_ of the sample, A_c1_ and A_c2_ were, respectively, the OD_536_ of c1 and c2. Two technical replicates were carried out for each biological replicate.

Survival to oxidative stress was assessed by exposing cells (collected in E1, 5 h; S1, 12 h; E2, 29 h; S2, 42 h) to H_2_O_2_ (50 mM) and pyrogallol (300 mM) for 30 min at 37°C; tolerance of heat stress was evaluated by incubating cells at 55°C for 30 min; survival to freezing and freeze-drying stresses was measured on the frozen and freeze-dried cells (see Ianniello et al., 2016 for processes) after 30, 60, and 90 days of storage at -20°C. For each stress, the number of healthy, damaged, viable but non-cultivable (VBNC) and dead cells were estimated using different WMB agar (WMA) media incubated for 48 h at 37°C in anaerobiosis. Specifically, the number of cultivable cells (healthy) was recovered on WMA pH 6.8; the number of VBNC cells was calculated as difference between the number of cultivable cells on WMA pH 6.8 with 0.05% w/v cysteine (scavenger of reactive oxygen species) and the number of cultivable cells on WMA pH 6.8; the number of damaged cells was calculated as difference between the number of cultivable cells on WMA pH 6.8 and the number of cultivable cells on WMA pH 5.5 (sub-lethal acid stress); the number of dead cells was calculated as difference between the number of cultivable non-stressed cells (control) and the number of cultivable healthy+VBNC cells. Two technical replicates were carried out for each biological replicate.

### Quantification of Relative Gene Expression Using Real-Time PCR

The relative gene expression of acetaldehyde dehydrogenase/alcohol dehydrogenase (*adha*), acetate kinase (*ack*), α-acetolactate decarboxylase (*ald*), acetolactate synthase (*als*), L-lactate dehydrogenase (*ldh*), oxaloacetate decarboxylase (*oad*), phosphate acetyltransferase (*pta*), pyruvate carboxylase (*pyc*), pyruvate dehydrogenase (*pdh*), pyruvate formate lyase (*pfl*), and pyruvate oxidase (*pox*) was evaluated in anaerobic and respirative growing cells, collected in both exponential phases (E1, 5 h; E2, 29 h). Glyceraldehyde-3-phosphate dehydrogenase (*gapdh*) was selected as suitable housekeeping gene based on the results of Ianniello et al. (2016). Primers (Supplementary Table 1) were designed with Primer Express software 3.0 (Applied Biosystems, Concord, ON, Canada), using as template the gene sequences retrieved from *L. casei* N87 genome (Zotta et al., 2016). RNA isolation, cDNA synthesis and amplification program were performed using the protocols optimized and described in Ianniello et al. (2016). Quantitative Real-Time PCR (qRT-PCR) was performed in a StepOne^TM^ real-time PCR instrument (Applied Biosystems, Thermo Fisher Scientific, Waltham, MA, United States) using a SYBR Green master mix (Qiagen, Toronto, ON, Canada) and an amplification program that included 1 cycle at 95°C for 5 min, 40 cycles at 95°C for 30 s and 60**°**C for 30 s, with a melting curve of 95°C for 15 s, 60°C for 1 min and 95°C for 15 s (ramping rate 0.3°C/s).

The relative expression of all genes was estimated according to the comparative ΔΔCt method (Pfaffl, 2001), using glyceraldehyde-3-phosphate dehydrogenase (*gapdh*) as reference gene and the first exponential phase (E1) of anaerobic cultivation as reference growth condition. Reaction mixtures without cDNA template were used as negative controls. Two technical replicates of the gene expression analysis were carried out for each growth condition and biological replicate.

### Reagents, Culture Media, and Ingredients

Unless otherwise stated all reagents were obtained from Sigma-Aldrich (Milan, Italy), while culture media and ingredients were obtained from Oxoid Ltd. (Basingstoke, Hampshire, United Kingdom).

### Statistical Analysis

All statistical analyses (one-way analysis of variance, multiple mean comparison with Tukey’s HSD *post hoc* test) and graphs were performed using Systat 13.0 for Windows (Systat Software Inc., San Jose, CA, United States).

## Results

### Anaerobic and Respiratory Growth of *Lactobacillus casei* N87 in Whey Permeate Medium

**FIGURE 1:**
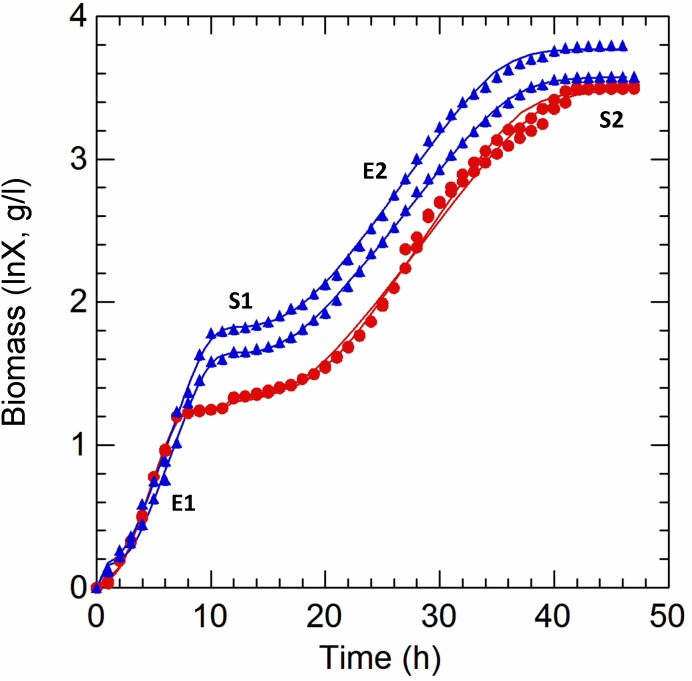
Kinetics of growth of *Lactobacillus casei* N87 cultivated in whey permeate medium. X axis: time (h) of cultivation. Y axis: ln-transformed biomass values (g/l). Red curves: AN, anaerobic cultivation. Blue curves: respiratory cultivation. Data obtained from growth modeling with the dynamic model of Baranyi and Roberts (1994), by using DMFit v. 3.5 package, were retrieved and used to plot the fit of growth curves (continuous lines). Experimental data (symbols) were merged and used for comparison. The two biological replicates were reported for each cultivation (AN, RS). Growth phases: E1 and E2, first and second exponential growth phases; S1 and S2, first and second stationary growth phases. All analyses described in Materials and Methods section were carried out on samples withdrawn in E1 (5 h), S1 (12 h), E2 (29 h), and S2 (42 h).

Cultivation of *Lactobacillus casei* N87 in whey permeate medium (WPM) induced a diauxic growth, in both anaerobic and respiratory conditions (Figure 1). Because of this, two exponential (E1, E2) and two stationary (S1, S2) phases were identified for each cultivation, and the parameters of first (E1+S1) and second (E2+S2) growth curves were estimated with the primary dynamic model of Baranyi and Roberts (1994). The results are shown in Table 1. The type of cultivation (RS vs. AN) did not affect (Tukey’s HSD, *p* ≤ 0.01) the maximum specific growth rate (μ_max_), but the values calculated in E2 were half of those estimated in E1, for both anaerobic and respiratory conditions. Respiration did not affect the lag phase in the primary growth, but reduced the diauxic lag period. Biomass (g/l) significantly accumulated during the second growth (E2+S2), but it was not appreciably boosted by supplemented aerobiosis. As expected, cells cultivated under respiration were able to consume oxygen (Table 1).

**Table 1:** Estimated growth parameters of *Lactobacillus casei* N87 cultivated in whey permeate medium.

Cond^a^	Phase^b^	dO_2_^c^	Lag^d^	μ_max_^e^	X_max_^f^	Res^g^
AN	E1	0	2.02 ± 0.08	0.65 ± 0.00	0.21 ± 0.00	≥180
AN	S1	0			0.49 ± 0.00	≥180
AN	E2	0	6.51 ± 0.71 ^§^	0.31 ± 0.03 ^§^	2.20 ± 0.03 ^§^	≥180
AN	S2	0			5.65 ± 0.07^‡^	≥180
RS	E1	60	2.68 ± 0.33	0.61 ± 0.02	0.20 ± 0.01	45 ± 7^∗^
RS	S1	60			0.71 ± 0.06^∗^	73 ± 4^∗^
RS	E2	60	3.61 ± 0.30	0.29 ± 0.02 ^§^	2.64 ± 0.06^§^	64 ± 6^∗^
RS	S2	60			6.05 ± 0.21^‡^	≥ 180^‡^


*Mean values ± standard errors of two biological replicates are shown.*

*^a^Growth conditions: AN, anaerobiosis; RS, respiration.*

*^b^Growth phase: first and second exponential (E1, E2) and stationary (S1, S2) phases.*

*^c^dO_*2*_: dissolved oxygen concentration (60%).*

*^d^Lag: lag phase (h; first growth) and diauxic lag phase (h; second growth).*

*^e^μ_max_: maximum specific growth rate (h^-1^); R^2^ of fit ranged from 0.995 to 0.999.*

*^f^ X _max_: maximum biomass production (g/l).*

*^g^Res: Oxygen consumption expressed as time (min) of resazurin discoloration; values ≥ 180 indicate no oxygen uptake.*

*^∗^Significant differences (Tukey’s HSD, p ≤ 0.01) between AN vs. RS within the same growth phases.*

*^§^ Significant differences (p ≤ 0.01) between E1 vs. E2 within the same growth condition.*

*^‡^ Significant differences (p ≤ 0.01) between S1 vs. S2 within the same growth condition.*

### Sugar Consumption and Production of Metabolites

The sugar consumption and the production of main metabolites are reported in Table 2. Lactose (111.0 ± 0.74 mM) and the residual galactose (18.6 ± 0.37 mM) were completely consumed in both anaerobic and respiratory cultivation. However, at the end of first growth (S1) only 18% of the total lactose was used by *L. casei* N87. The concentrations of glucose (0.05 g/l) and citrate (0.04 g/l) were very low in un-inoculated WPM and no consumption was observed during growth.

**Table 2:** Consumption of sugars and production of the main metabolites in *Lactobacillus casei* N87 cultivated in whey permeate medium.

Cond^a^	Lactose^b^	Galactose^b^	Lactate^c^	% (L-)^c^	Acetate^d^	Ethanol^e^	Diacetyl^f^	Acetoin^g^	Est pyr^h^	% Pyr^i^
AN_E1	13.4 ± 0.37	2.4 ± 0.37	27.0 ± 0.57	97.6 ± 1.12	9.3 ± 0.32	0.0 ± 0.00	0.1 ± 0.00	0.6 ± 0.01	58.1 ± 0.74	53.6 ± 1.58
AN_S1	20.5 ± 0.11	16.3 ± 0.26	34.9 ± 1.32	98.7 ± 1.54	12.7 ± 0.22	0.0 ± 0.00	0.1 ± 0.00	0.4 ± 0.02	114.4 ± 0.96	69.5 ± 1.41
AN_E2	64.7 ± 1.59§	17.0 ± 0.11§	209.5 ± 1.77§	90.8 ± 0.31§	22.2 ± 0.42§	0.0 ± 0.00	0.0 ± 0.00	0.3 ± 0.05§	292.7 ± 6.59§	28.4 ± 1.01§
AN_S2	110.1 ± 1.60‡	18.4 ± 0.47	331.4 ± 1.29‡	91.0 ± 0.33‡	22.4 ± 0.48‡	0.0 ± 0.00	0.0 ± 0.01	0.2 ± 0.02‡	477.2 ± 7.34‡	30.5 ± 1.34‡
RS_E1	15.2 ± 0.00	4.5 ± 0.36	25.7 ± 1.21	98.2 ± 0.36	9.8 ± 0.64	0.0 ± 0.00	1.3 ± 0.03*	0.7 ± 0.02*	69.7 ± 0.73	63.1 ± 1.34
RS_S1	20.1 ± 0.11	16.6 ± 0.15	33.6 ± 1.01	95.8 ± 0.59	18.1 ± 0.30	0.0 ± 0.00	1.4 ± 0.02*	1.4 ± 0.03*	113.8 ± 0.74	70.5 ± 1.08*
RS_E2	67.5 ± 1.04§	17.4 ± 0.48§	162.5 ± 3.82*§	89.3 ± 0.85§	42.4 ± 0.73*§	0.0 ± 0.00	1.8 ± 0.04*§	5.8 ± 0.02*§	304.8 ± 5.11§	46.7 ± 2.15§
RS_S2	110.8 ± 0.52‡	18.4 ± 0.29	299.8 ± 3.81*‡	90.2 ± 0.13‡	92.5 ± 2.77*‡	0.0 ± 0.00	2.3 ± 0.09*‡	12.0 ± 0.07*‡	480.1 ± 2.66‡	37.5 ± 1.14‡
WPM	111.0 ± 0.74	18.6 ± 0.37	3.3 ± 0.01	–	0.3 ± 0.08	10.4 ± 0.05	0.0 ± 0.00	0.2 ± 0.02	–	–


*Mean values ± standard errors of two biological replicates are shown.*

*^a^Growth conditions: AN, anaerobiosis; RS, respiration; first and second exponential (E1, E2) and stationary (S1, S2) phases; WPM, un-inoculated whey permeate medium.*

*^b^Consumed lactose (L_WPM_-L; mM) and galactose (G_WPM_-G; mM), calculated as difference between un-inoculated and fermented WPM.*

*^c^Production of DL-lactic acid (DL-DL_WPM_; mM) and percentage of L-isomer (% L-).*

*^d^Production of acetic acid (A-A_WPM_; mM).*

*^e^Production of ethanol (E_WPM_-E; mM); the presence of ethanol in un-inoculated WPM was due to the preparation of growth medium.*

*^f^Production of diacetyl (D-D_WPM_; mM).*

*^g^Production of acetoin (Ac-Ac_WPM_; mM).*

*^h^The potential pyruvate concentration (mM), was estimated by measuring the consumed mmol of lactose and galactose (^b^).*

*^i^Percentage of estimated pyruvate concentration (mM) rerouted away from lactate production (mM) and available for the conversion in other products; values were calculated as the % of estimated mmol of pyruvate not converted into mmol of lactate.*

*^∗^Significant differences (Tukey’s HSD, p ≤ 0.01) between AN vs. RS within the same growth phases.*

*^§^ Significant differences (p ≤ 0.01) between E1 vs. E2 within the same growth condition.*

*^‡^Significant differences (p ≤ 0.01) between S1 vs. S2 within the same growth condition.*

DL-lactate was the main metabolite accumulated at the end (S2) of anaerobic and respiratory cultivations, but its production during the first growth was significantly lower. Respiration reduced the amounts of DL-lactate in all growth phases, although the greatest effect was observed in the second curve. L-lactate was the main isomer produced in both anaerobic and respiratory conditions (Table 2), although its percentage was slightly lower during the second growth (E2, S2). Acetate was found in all supernatants, and respiration significantly increased its production (up to 4 times higher in S2). Ethanol was found in un-inoculated WPM since it was used for growth medium preparation, but no further production from anaerobic or respiratory cells was observed.

### Production of Volatile Organic Compounds

The Volatile Organic Compounds (VOCs) produced by *L. casei* N87 during anaerobic and respiratory growth in WPM were evaluated by SPME-GC/MS. Concentrations of diacetyl and acetoin were quantified and reported in Table 2. Diacetyl was not found in supernatants of anaerobic fermentation, but it accumulated under respiratory growth (up to 200 mg/l in the supernatants collected in S2). Similarly, the acetoin production was significantly boosted under aerobic regime (up to 1.02 g/l the supernatants collected in S2).

**FIGURE 2:**
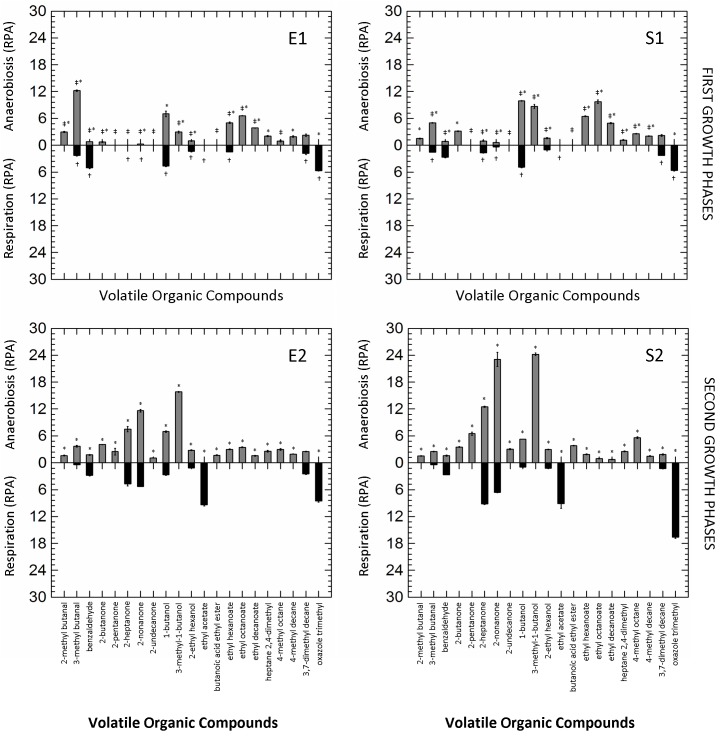
Mirror plots of Relative Peak Area (RPA) of Volatile Organic Compounds (VOCs) produced by *Lactobacillus casei* N87 cultivated in whey permeate medium in anaerobic (gray bars) and respirative (black bars) conditions. E1 and E2: first and second exponential growth phases. S1 and S2: first and second stationary growth phases. Mean values ± standard errors of two biological replicates are shown. ^‡^Significant differences (Tukey’s HSD, *p* ≤ 0.01) in VOC production between anaerobic cells collected in first and second exponential phase (E1 vs. E2) or in first and second stationary phase (S1 vs. S2). **^†^**Significant differences (*p* ≤ 0.01) in VOC production between respirative cells collected in first and second exponential phase (E1 vs. E2) or in first and second stationary phase (S1 vs. S2).^∗^Significant differences (*p* ≤ 0.01) in VOC production between anaerobic and respirative cells within the same growth phases.

The other 21 VOCs with a Relative Peak Area (RPA; semi-quantitative parameter) ≥ 2.0-fold-change in at least one of the tested conditions were selected and retained for further analysis. The production of VOCs in the different growth conditions (RS vs. AN) and phases (first vs. second curves) is shown in Figure 2. Respiration significantly reduced the number of VOCs in all growth phases, while the prolonged cultivation (E2, S2) clearly increased their accumulation in both anaerobic and respirative cultures. Benzaldehyde, ethyl acetate and oxazole trimethyl were the marker compounds (mainly or exclusively present) of respirative cells, while different ketones (e.g., 2-pentanone, 2-heptanone, 2-nonanone) and secondary alcohols (e.g., 1-butanol, 3-methy-1-butanol) were remarkably produced during anaerobic growth. Other compounds (e.g., 2-methyl butanal, 2-butanone, 3-methyl-1-butanol, butanoic acid ethyl ester, ethyl hexanoate, ethyl octanoate, ethyl decanoate) were exclusively produced by anaerobic cells during all cultivation.

### Relative Expression of Genes Involved in the Main Metabolic Pathways of *L. casei* N87

**FIGURE 3:**
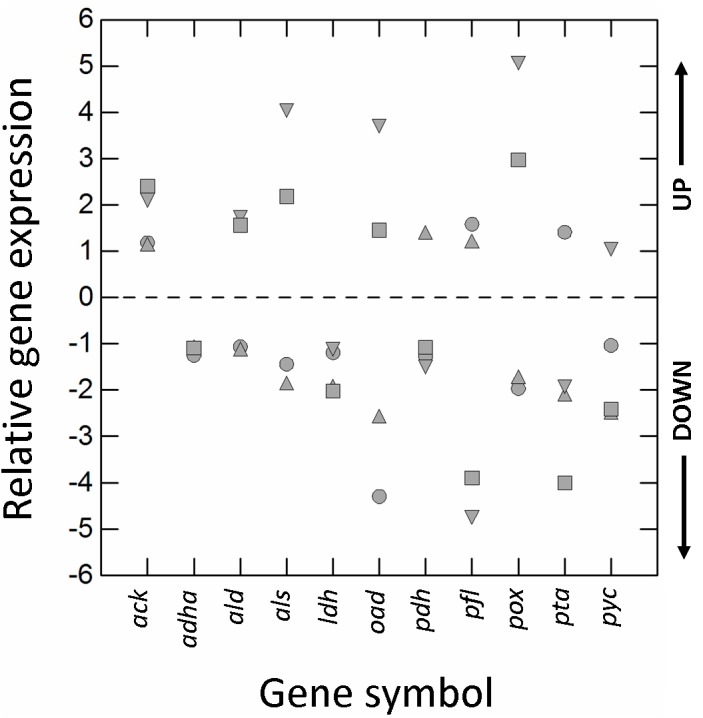
Relative gene expression (RGE) of acetaldehyde dehydrogenase/alcohol dehydrogenase (*adha*), acetate kinase (*ack*), α-acetolactate decarboxylase (*ald*), acetolactate synthase (*als*), L-lactate dehydrogenase (*ldh*), oxaloacetate decarboxylase (*oad*), pyruvate dehydrogenase (*pdh*), pyruvate formate lyase (*pfl*), pyruvate oxidase (*pox*), phosphate acetyltransferase (*pta*), and pyruvate carboxylase (*pyc*) genes of *Lactobacillus casei* N87 grown in whey permeate. Mean values of 2 biological and 2 technical replicates are shown. Symbols: circles, ratio between RGE (E2 vs. E1) measured in cells cultivated anaerobically; up-triangles, ratio between RGE (E2 vs. E1) measured in cells cultivated under respiration; down-triangles, ratio between RGE measured in respirative (RS) and anaerobically (AN) growing cells collected in the first exponential phase (E1); squares, ratio between RGE measured in respirative (RS) and anaerobically (AN) growing cells collected in the second exponential phase (E2). UP, up-regulation (positive fold-change); DOWN, down-regulation (negative fold-change). Values ≥ and ≤ than ± 1.5-fold-change indicate significant differences (Tukey’s HSD, *p* ≤ 0.01) in RGE compared to the reference growth conditions (AN, E1).

The type of cultivation (RS vs. AN) and growth phase (E2 vs. E1) significantly affected (±1.5-fold-change; *p* ≤ 0.01) the transcription of almost all genes (Figure 3). Respiration increased the relative expression of pyruvate oxidase (*pox*), acetolactate synthase (*als*) and oxaloacetate decarboxylase (*oad*), but decreased that of L-lactate dehydrogenase (*ldh*), pyruvate formate lyase (*pfl*), pyruvate carboxylase (*pyc*), and phosphate acetyltransferase (*pta*).

Genes encoding for acetoin reductase (*ar*), butanediol dehydrogenase/diacetyl reductase (*bdh*), citrate synthase (*cs*), NADH-dependent lactate oxidase (*lox*), and malate dehydrogenase (*mae/mle*) were not considered in this analysis because the genome of *L. casei* N87 does not harbor the above sequences. Moreover, although *L. casei* N87 has the citrate lyase (*cl*) complex (*α*-subunit/citrate CoA-transferase; *β*-subunit/citryl-CoA lyase; *γ*-subunit/acyl carrier protein), the relative expression of *cl* coding genes were not investigated since no amount of citrate was added to WPM and that already present (only 0.04 g/l) was not consumed.

**FIGURE 4:**
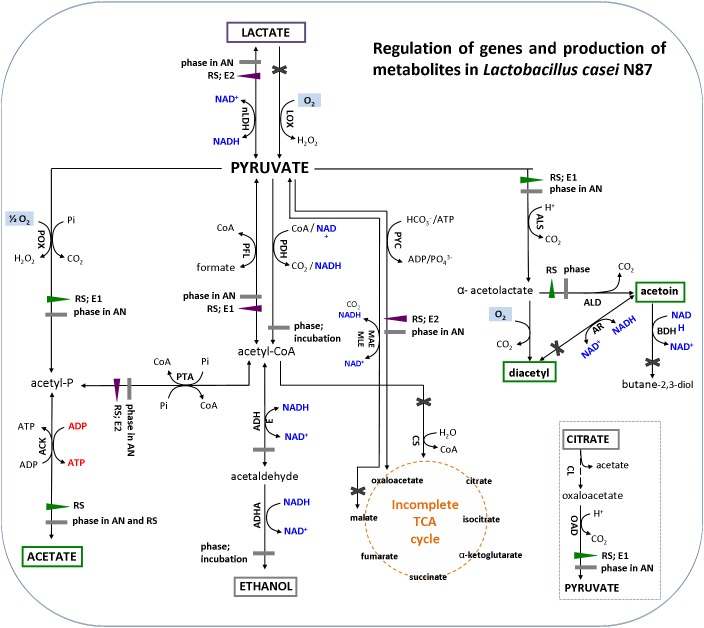
Metabolic pathways for pyruvate conversion in *Lactobacillus casei* N87 cultivated in whey permeate medium (figure was adapted from the Figure 1 of Zotta et al., 2017a). Blue font: formation of reduced (NADH) or oxidized (NAD^+^) cofactors; red font: ATP generation; pale blue box: reaction requiring oxygen. Enzymes in metabolic pathways are indicated as follow (alphabetic order): ACK, acetate kinase; ADHA/ADHE, acetaldehyde dehydrogenase/alcohol dehydrogenase; ALD, α-acetolactate decarboxylase; ALS, α-acetolactate synthase; AR, acetoin reductase; BDH, butanediol dehydrogenase/diacetyl reductase; CL, citrate lyase; CS, citrate synthase; LDH, lactate dehydrogenase; LOX, lactate oxidase; MAE/MLE, malate dehydrogenase; OAD, oxaloacetate decarboxylase; PDH, pyruvate dehydrogenase; PFL, pyruvate formate lyase (PFL); pyruvate oxidase (POX); PTA, phosphotransacetylase; PYC, pyruvate carboxylase. Regulation of metabolic pathways: violet left- or down-triangle, down-regulation of gene expression; green right- or up-triangle, up-regulation of gene expression; gray rectangle, no significant regulation of gene expression; black cross, genes not found in the genome of *Lactobacillus casei* N87. Factors (AN, anaerobiosis; RS, respiration; E1 and E2, respectively, first and second exponential growth phases) affecting the relative gene expression are annotated next to regulation symbols. Metabolites in colored box have been measured experimentally in this work: green box, over-production in respirative condition; purple box, over-production in anaerobic condition; gray box, no significant differences between anaerobic and respirative conditions.

The results of gene expression reflected the quantification of main metabolites (Figure 4). The amounts of acetate, acetoin and diacetyl (green box) were significantly higher in respirative conditions, consistently with up-regulation of *pox*-*ack* (acetate pathway) and *als*-*ald* (acetoin and diacetyl pathways) genes. Production of DL-lactate (purple box) was lower under respiration, because of down-regulation of *ldh*. The expression of alcohol dehydrogenase (*adh*) was not affected by atmosphere of incubation and growth phases and, then, no production of ethanol (gray box) was observed in both anaerobic and respirative growth.

### Antioxidant Capability and Stress Tolerance

The respiratory cells exhibited a major antioxidant activity (from 87 to 96%; *p* ≤ 0.01) compared to those grown anaerobically (Supplementary Figure 1). Cells collected in the second growth phases (E2, S2), moreover, removed hydroxyl radicals to a significantly greater extent.

**FIGURE 5:**
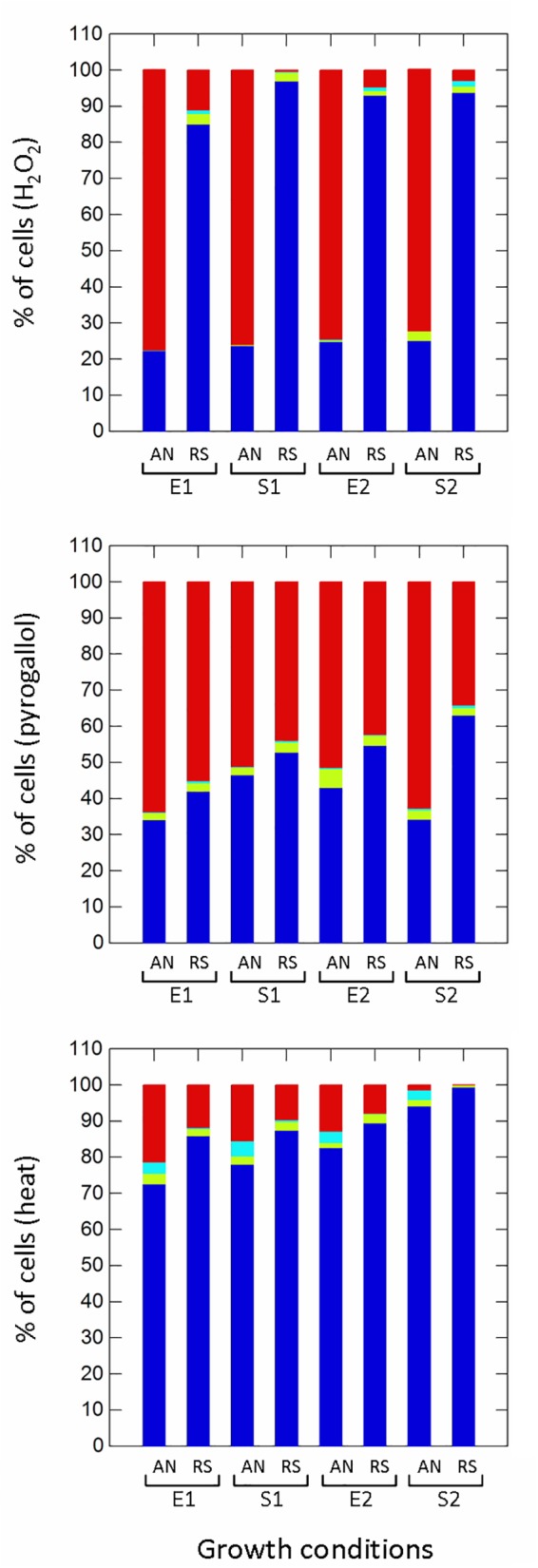
Survival of *Lactobacillus casei* N87 to oxidative (hydrogen peroxide, pyrogallol) and heat stresses. AN: anaerobiosis; RS: respiration. E1 and E2: first and second exponential growth phases; S1 and S2: first and second stationary growth phases. Color bars: blue, % of cultivable healthy cells; light blue, % of VBNC cells; green bars, % of damaged cells; red bars, % of dead cells. Mean values of 2 biological and 2 technical replicates were used.

**FIGURE 6:**
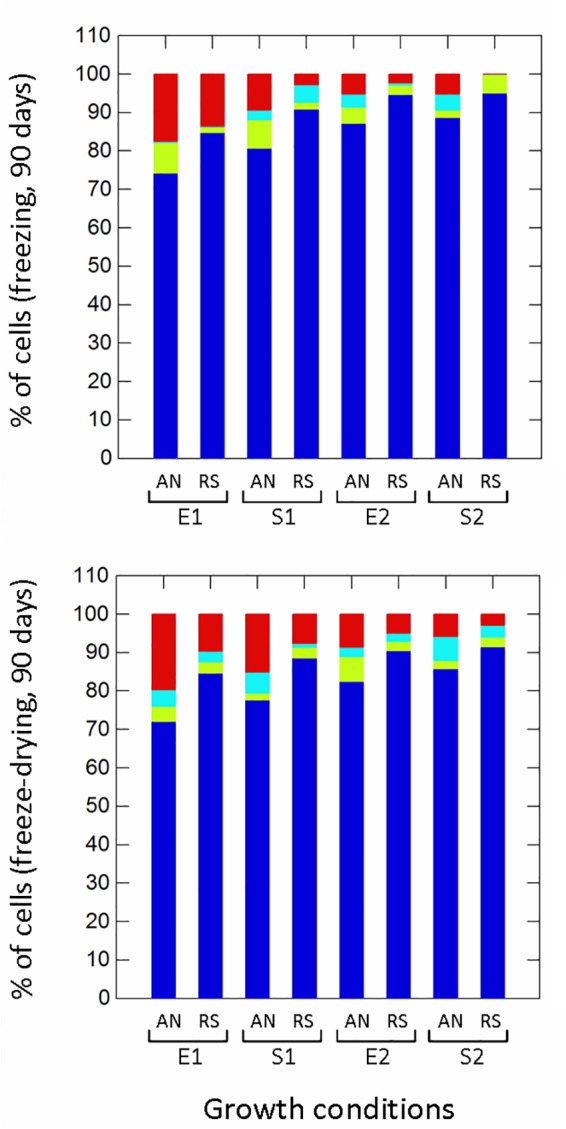
Survival of *Lactobacillus casei* N87 to freezing and freeze-drying processes after 90 days of storage at -20°C. AN, anaerobiosis; RS, respiration. E1 and E2, first and second exponential growth phases; S1 and S2, first and second stationary growth phases. Color bars: blue, % of cultivable healthy cells; light blue, % of VBNC cells; green bars, % of damaged cells; red bars, % of dead cells. Mean values of 2 biological and 2 technical replicates were used.

Respiration significantly (*p* ≤ 0.01) improved the survival to H_2_O_2_ (non-radical reactive oxygen species) compared to anaerobic cultivation (Figure 5), while growth phases affected the proportion of healthy, damaged, VBNC and dead cells only in aerobic conditions. Aerobic growth increased the survival to pyrogallol (superoxide radical generator), although to a lesser extent compared to H_2_O_2_ exposure (Figure 5). Respirative stationary cells were more resistant than exponential ones, while the prolonged anaerobiosis (S2) significantly reduced the number of survivors. Respiration also improved the robustness to heat stress (Figure 5). The prolonged cultivation, however, increased tolerance in both anaerobic and respiratory cultures, and at the end of growth (S2) the number of healthy cells was significantly high (94% in anaerobiosis, 99% in respiration). The type of cultivation affected the survival of frozen (Supplementary Figure 2 and Figure 6) and freeze-dried (Supplementary Figure 3 and Figure 6) cells mainly after 90 days of storage (Figure 6). Respiratory growth improved the survival to long-term freezing and freeze-drying in all phases and, with few exceptions, reduced the number of damaged and VBNC cells compared to anaerobic cultivation.

## Discussion

In this study, a WMP was used as low-cost substrate to cultivate *L. casei* N87 in anaerobic and respiratory conditions. Differently from the previous study of Ianniello et al. (2016), the volatile organic profile and the transcription of genes involved in the main metabolites production were investigated. Growth performances and stress robustness were also compared.

### Diauxic Growth and Sugar Consumption in Whey Permeate Medium

The growth of *L. casei* N87 in WPM exhibited a diauxic trend in both anaerobic and respiratory conditions. This behavior was not observed in WMB, suggesting that diauxie was induced by the presence of multiple substrates (i.e., lactose and galactose). The μ_max_ measured during the first growth in WPM were similar to those reached in WMB, but the biomass production was significantly lower. On the other hand, only 18% of lactose was consumed up to S1. Lag phases in WPM were also much longer than those calculated in WMB. Although the secondary growth in WPM was not comparable with cultivation in WMB, the final biomass reached in the low-cost substrate was higher than that gained in complex medium.

The factors (e.g., carbon catabolite repression, CCR; phosphorylation of HPr; carbohydrate phosphotransferase system, PTS) affecting the diauxic growth of *L. casei* on different PTS (e.g., glucose, lactose) and non-PTS (e.g., maltose, ribose) sugars was investigated by Viana et al. (2000) under anaerobic conditions. Jyoti et al. (2003), moreover, demonstrated a diauxic growth on multiple substrates (i.e., glucose+ citrate; glucose + lactate) also in the genetically related species *L. rhamnosus*. On the contrary, Rosa et al. (2013) found that *L. acidophilus* grown on a whey-based medium did not exhibit a diauxic pattern, suggesting that the lactose was hydrolyzed into glucose and galactose and both sugars were utilized simultaneously.

Our results demonstrated that diauxie was independent of the atmosphere of incubation, since a diauxic period was observed in both anaerobic and respiratory conditions. The consumption of lactose and of residual galactose, in fact, was similar in both growth conditions.

*L. casei* N87 consumed both lactose and residual galactose present in WP. The genome of *L. casei* N87 harbors the sequences encoding for lactose-specific and galactose-specific phosphotransferase (PTS) systems (Lac-PTS and Gal-PTS), and for the enzymes involved in lactose conversion (6-P-*β*-galactosidase, LacG) and galactose utilization via tagatose-6-P pathway (i.e., galactose-6-P isomerase, tagatose-6-P kinase, tagatose-1,6-P aldolase), but does not possess the lactose permease and galactose permease genes. Therefore, as previously suggested by Wu and Shah (2017), we assumed that the membrane PTS and tagatose-6-P pathway mediate the lactose and galactose metabolism in *L. casei* N87. Our results, moreover, demonstrated that *L. casei* N87 was able to consume lactose without galactose accumulation. Because of this, the strain could be exploited as adjunct culture for the production of dairy products (e.g., cheeses, yogurt) with low lactose and galactose content that are suitable for lactose intolerant and galactosemic people.

### Production of Acetoin and Diacetyl Under Respiratory Condition

The production of acetoin and diacetyl (ALS-driven metabolites) was higher under respirative growth. In the last decades, several mutagenesis approaches (Platteeuw et al., 1995; Benson et al., 1996; Swindell et al., 1996; López de Felipe et al., 1998; Curic et al., 1999; Gosalbes et al., 2000; Hugenholtz et al., 2000; Kleerebezem et al., 2000; Oliveira et al., 2005; Nadal et al., 2009) based on the genetic manipulation of lactate dehydrogenase, α-acetolactate synthase, α-acetolactate decarboxylase and H_2_O-forming NADH-oxidase have been used to promote the accumulation of these compounds in some LAB. Many authors, moreover, demonstrated that unsupplemented and/or heme-supplemented aerobiosis might boost the production of acetoin and diacetyl in several wild-type (Koebmann et al., 2008; Pedersen et al., 2008; Zhao et al., 2013; Cesselin et al., 2018) and mutant (López de Felipe et al., 1998; Monnet et al., 2000; Liu et al., 2016a) strains of *Lc. lactis*.

More recently, the increased production of diacetyl and/or acetoin has been verified also in some oxygen-tolerant lactobacilli cultivated under respiration (Ianniello et al., 2015, *L. rhamnosus* N132 in complex synthetic medium; Reale et al., 2016b, *L. casei* N87 and N2014 in Cheddar-type cheeses; Zotta et al., 2017b, *L. casei* N87 in chemically defined medium). The concentrations (1.06 g/l acetoin; 0.23 g/l diacetyl; 1.29 g/l acetoin+diacetyl) reached with respirative cells of *L. casei* N87 were comparable with those obtained by Nadal et al. (2009) with a *L. casei* BL23 mutant cultivated in WPM under batch (1.0 g/l acetoin+diacetyl, pH 6.5) and fed-batch (1.2 g/l acetoin+diacetyl, pH 6.5; 1.4 g/l acetoin+diacetyl, pH 5.5) conditions. As the genome of *L. casei* N87 does not contain acetoin reductase gene (conversion of acetoin to diacetyl), diacetyl was produced exclusively via non-enzymatic oxidative decarboxylation of α-acetolactate. Moreover, since *L. casei* N87 also lacks the butanediol dehydrogenase (*bdh*) gene, and the transcription of α-acetolactate decarboxylase (*ald*) increased with oxygen supplementation, acetoin accumulated more than diacetyl under respiratory growth.

### Production of Novel Volatile Compounds Under Respiratory Conditions

The type of cultivation affected others VOCs in *L. casei* N87. Benzaldehyde (almond-like flavor) and ethyl acetate (fruity-like flavor) were the main products of respirative cultures grown in WP. Benzaldehyde, together with vanillin, is one of the most important compounds used in food, beverage and fragrance industries; it is generally obtained via chemical synthesis or extracted from fruit kernels, with possible release of toxic by-products and, therefore, its production by respiratory phenotypes may be an interesting biotechnological alternative to avoid the formation of harmful products. Ethyl acetate occurs in several cheese varieties (Liu et al., 2004) and, therefore its production through respiration-based technology may be of practical relevance in dairy sector.

2,4,5-trimethyl oxazole (nutty-like flavor), an aroma compound resulting from the reactions between diacetyl and aminoacids (Marchand et al., 2011), was also increased by prolonged aerated growth. 2,4,5-trimethyl oxazole contributes to the aroma profile of roasted foods (e.g., potato, peanuts, coffee), but is also a powerful odorant in wine (Marchand et al., 2000, 2011) and Chinese traditional vinegars (Yu et al., 2012; Zhu et al., 2016). As this compound has not been previously identified among the metabolites produced by respiration-competent LAB, this aspect deserves further investigation.

As already proven (Reale et al., 2016a,b cheddar-type cheeses; sourdoughs; Zotta et al., 2017b, chemically defined medium), respiratory cultivation reduced the content of some ketones resulting from lipid and/or protein oxidation as well as some hydrocarbons and branched-chain aldehydes. The latter compounds (e.g., 2-methyl butanal, 3-methyl butanal) play an important role in the aroma profile of many hard/semi-hard and surface-ripened cheeses, but their content, if high, may result in off-flavor for several soft cheeses. Because of this, several strategies, including the use of suitable starter and/or adjunct cultures or oxygen/oxidizing agents, have been proposed to control the production of this compounds in dairy products (Afzal et al., 2017). The respirative phenotype of *L. casei* N87, therefore, could be efficiently used to develop a balanced-volatile profile in several cheese varieties.

### Survival and Antioxidant Features of Respirative Cells

Our results confirmed that *L. casei* N87 exhibited a greater antioxidant capability and tolerance of oxidative stress when cultivated under respiration. *L. casei* N87 possess both heme- and Mn-dependent catalases that contribute to H_2_O_2_ degradation. Recently, Ricciardi et al. (2018) demonstrated that enzymatic activities and gene expression of both enzymes were significantly improved by aerobic condition and heme supplementation. On the contrary, *L. casei* N87 does not harbor a superoxide dismutase useful to inhibit the harmful effect of pyrogallol; the strain, however, exhibited a high survival to pyrogallol, suggesting that other mechanisms may reduce the formation of superoxide radicals. The survival of cells grown in WPM, however, was slightly lower than that estimated in *L. casei* N87 cultivated in WMB, although the results were not completely comparable as the cells were collected at different growth phases and physiological state.

To our knowledge, the effect of atmosphere of incubation on the tolerance to heat stress has been investigated only in *L. plantarum* (Zotta et al., 2012, 2013), demonstrating that survival to high temperatures was related to the interactions of several factors (e.g., presence of oxygen, temperature of cultivation, growth phase, gene regulation). In this study, on the contrary, the respirative cultivation increased the number of survivors regardless of growth phases (exponential, stationary) and curves (primary, secondary). Respiration conferred a greater resistance also to storage processes. The robustness of cold-starved, frozen and freeze-dried cells cultivated under respiration has been previously proven in at least some strains of *Lc. lactis*, *L. casei*, and *L. plantarum* (see Pedersen et al., 2012; Zotta et al., 2017a as review), suggesting that aerobic growth supplemented with hemin and menaquinone could be efficiently exploited to guarantee the viability and fitness of starter, adjunct and probiotic cultures.

## Conclusion

This study confirms that respiration-based technology coupled with cultivation on low-cost media may be exploited as natural strategy to produce competitive (increased biomass yield and stress robustness) and functional (control of desirable metabolites) starter and/or adjunct cultures.

However, although the production of important metabolites, such as diacetyl and acetoin, was demonstrated at both phenotypic and genetic level, further optimization of growth conditions (e.g., fed-batch and/or chemostat cultivations to reach high-cell density cultures, different dissolved oxygen concentrations and sugar content) are needed so that production of these compounds becomes higher and interesting for industrial-scale applications.

This study provided additional information on the activation and re-direction of pyruvate pathways under respiratory growth. With exception of very few papers that analyzed the changes in gene expression through global transcriptome approaches, in fact, most of data on aerobic and respiratory metabolism of LAB focused exclusively on the main genes affected by oxygen (e.g., pyruvate oxidase, cytochrome oxidase, NADH-oxidase, lactate dehydrogenase, pyruvate dehydrogenase), analyzing them often separately and not in relation to other genes involved in pyruvate conversion and metabolite production.

Finally, the potential of respirative phenotype of *L. casei* N87 as suitable cell factory for several food-related applications has been once more proven.

## Author Contributions

TZ, AR, and EP contributed the design of experimental activities, resources, supervision and validation of experimental activities, and writing – review and editing. TZ and EP performed data curation and analyses. RI, FB, AM, TZ, and AR contributed investigation, analyses and methodologies. TZ performed funding acquisition, project administration and writing the original draft.

## Conflict of Interest Statement

The authors declare that the research was conducted in the absence of any commercial or financial relationships that could be construed as a potential conflict of interest.
