# Supplementary material for: Effect of Respiratory Growth on the Metabolite Production and Stress Robustness of Lactobacillus casei N87 Cultivated in Cheese Whey Permeate Medium
Source: Front Microbiol. 2019 Apr 24;10:851. doi: 10.3389/fmicb.2019.00851 (PMC6491770; doi:10.3389/fmicb.2019.00851)
Supplement: Supplementary file 1 [file Data_Sheet_1.pdf]

Effect of respiratory growth on the metabolite production and stress robustness of *Lactobacillus casei* N87 cultivated in cheese whey permeate medium

Annamaria Ricciardi[1], Teresa Zotta[2,*], Rocco Gerardo Ianniello[1,#], Floriana Boscaino[2], Attilio Matera[1], Eugenio Parente[3]

[1] Scuola di Scienze Agrarie, Forestali, Alimentari e Ambientali, Università degli Studi della Basilicata, Potenza, Italy
[2] Istituto di Scienze dell'Alimentazione - CNR, Avellino, Italy
[3] Dipartimento di Scienze, Università degli Studi della Basilicata, Potenza, Italy

[*] **Corresponding author**
Dr. Teresa Zotta
Istituto di Scienze dell'Alimentazione – Consiglio Nazionale delle Ricerche, via Roma 64, 83100 Avellino, Italy
E-mail: teresa.zotta@isa.cnr.it
Tel.: +39-0825-299531
Fax: +39-0825-78158

[#] **Present address**
Dr. Rocco Gerardo Ianniello
Bonassisa Lab, Zona Industriale ASI, 71122 Foggia, Italy

Running title
Growth of *Lactobacillus casei* N87 in whey permeate

Supplementary Table 1: Sequences of forward (F) and reverse (R) primers used for quantification of relative gene expression in *Lactobacillus casei* N87.

Gene	Primer	Sequence	Amplicon
acetaldehyde/alcohol dehydrogenase (*adh*)	adh-N87-F<br>adh-N87-R	5'-ACGGCTGATGAAGACCTCGC-3'<br>5'-GCAAGTCAACCGTCCCCACA-3'	187 bp
acetate kinase (*ack*)	ack-N87-F<br>ack-N87-R	5'-GCAGCCTTGATGAACGGGGTA-3'<br>5'-GGCGTGCTGAGATCGGTTTC-3'	167 bp
alpha-acetolactate decarboxylase (*ald*)	ald-N87-F<br>ald-N87-R	5'-CCGCAACAGCCTCCTTATCCG-3'<br>5'-AGCCAAGAATGTGCCCACCC-3'	187 bp
acetolactate synthase (*als*)	als-N87-F<br>als-N87-R	5'-GGTACCGTTACTGCGAGGCTA-3'<br>5'-ACTGCCCACATCTACCGCA-3'	193 bp
L-lactate dehydrogenase (*ldh*)	ldh-N87-F<br>ldh-N87-R	5'-AGGTATTGCACAAGAAATCGGGA-3'<br>5'-GCCTGGCTTCTGAGGAGCAC-3'	181 bp
oxaloacetate decarboxylase (*oad*)	oad-N87-F<br>oad-N87-R	5'-CGATCAAAGACATGGCGGGC-3'<br>5'-CCCTGCACGCACCGATTCAA-3'	152 bp
phosphate acetyltransferase (*pta*)	pta-N87-F<br>pta-N87-R	5'-TTGACATTGATCCGCCACGAG-3'<br>5'-CGTCAAACTGCAACTCACCATCC-3'	150 bp
pyruvate carboxylase (*pyc*)	pyc-N87-F<br>pyc-N87-R	5'-CAGGTGCAAGCCAAGCCAGA-3'<br>5'-TGCGCCCTTGTCTTTCAGCA-3'	186 bp
pyruvate dehydrogenase (*pdh*)	pdh-N87-F<br>pdh-N87-R	5'-GCGTGAAGGAACCGACGT-3'<br>5'-ACCGATGCAATGATGGTCTC-3'	156 bp
pyruvate formate lyase (*pfl*)	pfl-N87-F<br>pfl-N87-R	5'-CCTTAGACAACATGGGTGCAGC-3'<br>5'-GCCATAATAGTCCGTGCCCC-3'	170 bp
pyruvate oxidase (*pox*)	pox-N87-F<br>pox-N87-R	5'-CGACTGCTGCCATGAACACC-3'<br>5'-GAATCGTCACCACCGCTACG-3'	171 bp
glyceraldehyde-3-P dehydrogenase (*gapdh*)	gadph-N87-F<br>gadph-N87-R	5'-GCACACCGAAAACAACCCTAGC-3'<br>5'-CGTTATCGTACCAAGCAACCGT-3'	161 bp

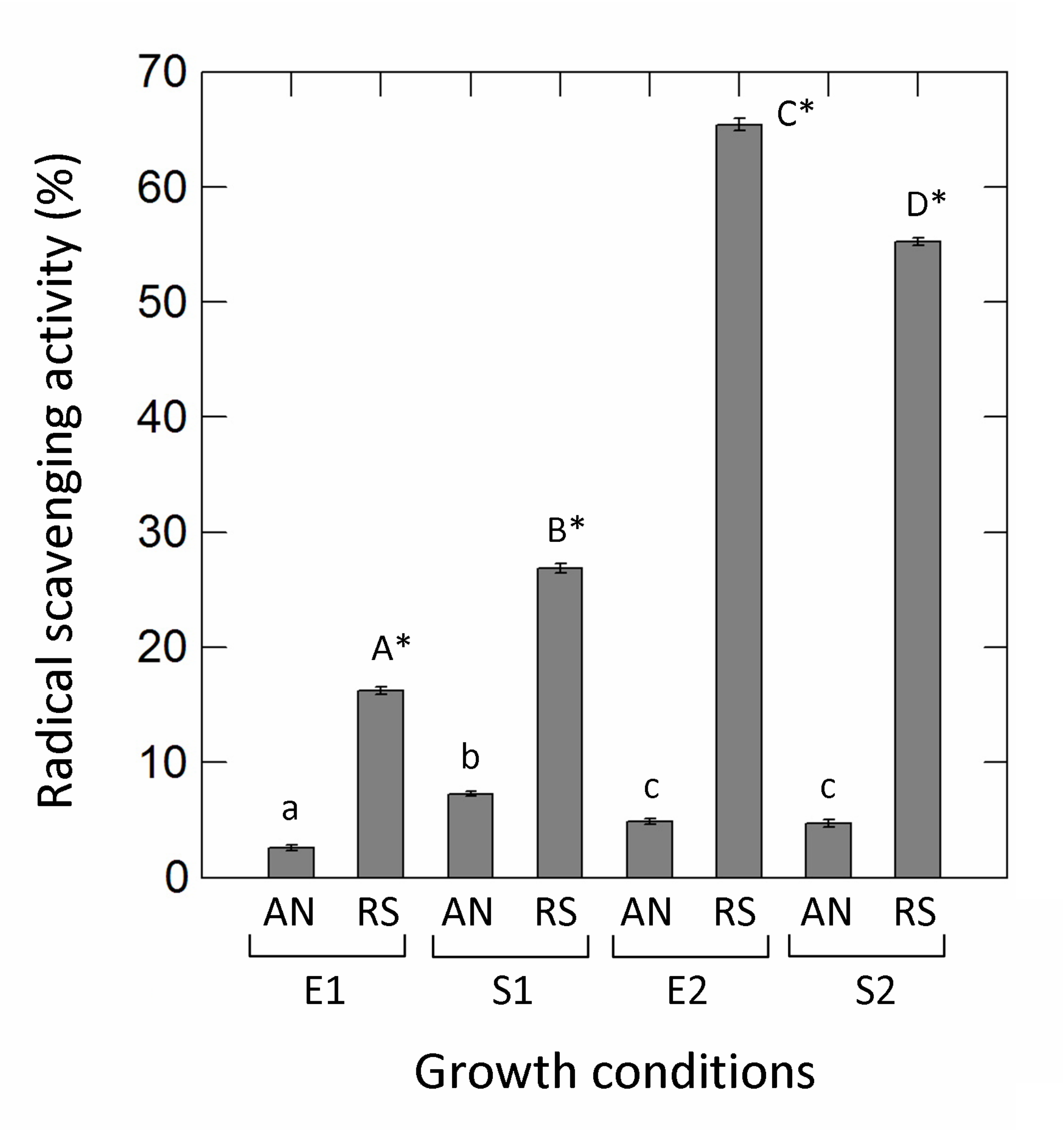

Supplementary Figure 1: Percentage of hydroxyl radical scavenging activity in *Lactobacillus casei* N87 cultivated in whey permeate medium. AN: anaerobiosis; RS: respiration. E1 and E2: first and second exponential growth phases; S1 and S2: first and second stationary growth phases. Lowercase letters on the bars indicate significant differences (Tukey's HSD, $p \leq 0.01$) in radical scavenging activity of cells cultivated anaerobically in the different growth phases (E1, S1, E2, S2). Uppercase letters on the bars indicate significant differences ($p \leq 0.01$) in radical scavenging activity of cells cultivated under respiration in the different growth phases (E1, S1, E2, S2); * indicates significant differences ($p \leq 0.01$) between anaerobically and respirative growing cells within the same growth phase.

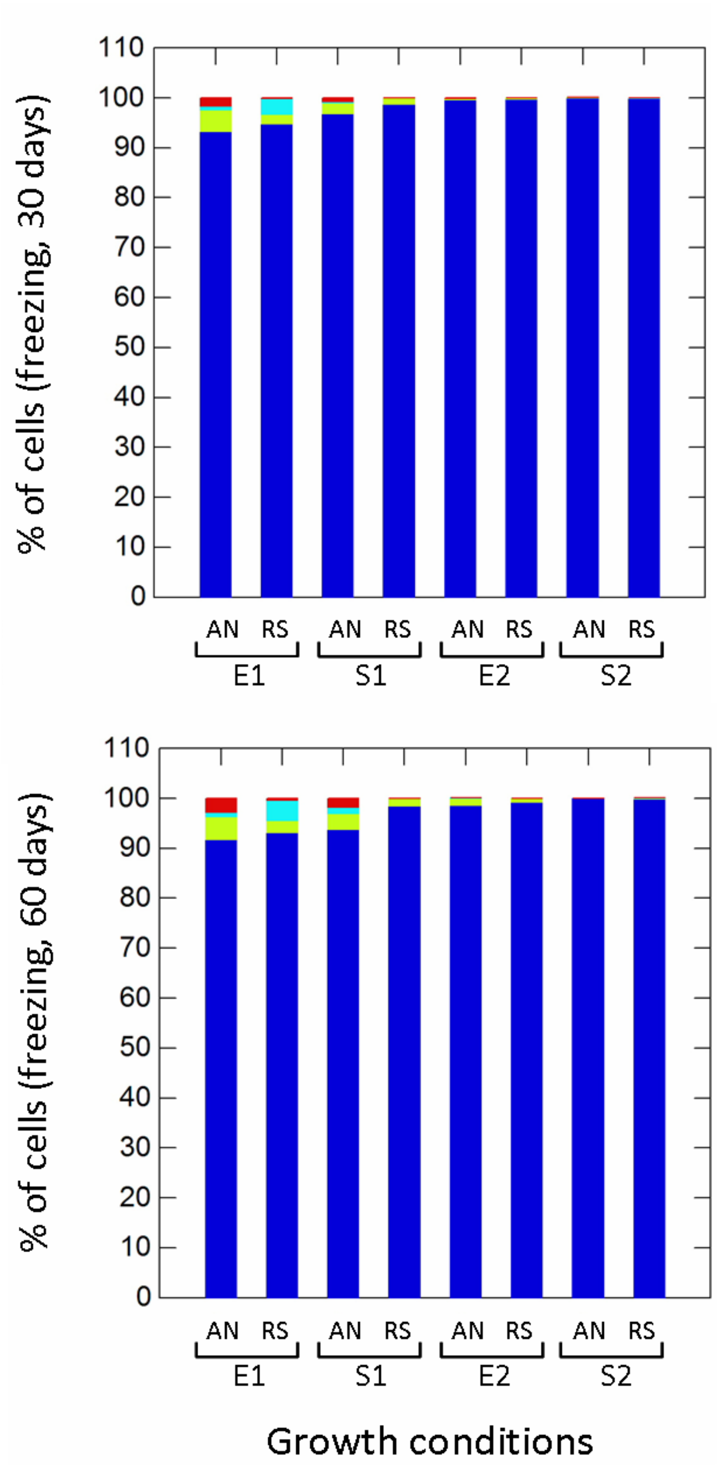

Supplementary Figure 2: Survival of *Lactobacillus casei* N87 to freezing process after 30 and 60 days of storage at -20°C. AN: anaerobiosis; RS: respiration. E1 and E2: first and second exponential growth phases; S1 and S2: first and second stationary growth phases. Color bars: blue, % of cultivable healthy cells; light blue, % of VBNC cells; green bars, % of damaged cells; red bars, % of dead cells.

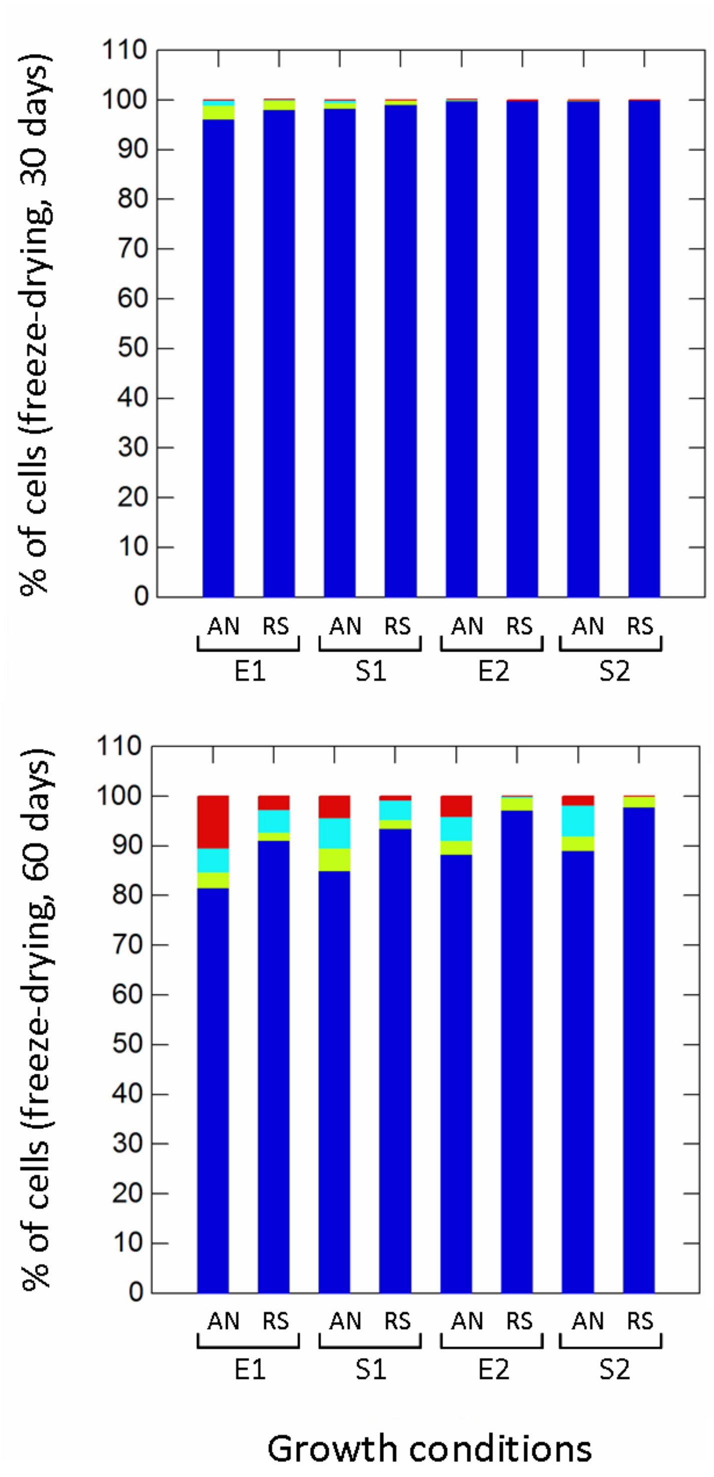

Supplementary Figure 3: Survival of *Lactobacillus casei* N87 to freeze-drying process after 30 and 60 days of storage at -20°C. AN: anaerobiosis; RS: anaerobiosis. E1 and E2: first and second exponential growth phases; S1 and S2: first and second stationary growth phases. Color bars: blue, % of cultivable healthy cells; light blue, % of VBNC cells; green bars, % of damaged cells; red bars, % of dead cells.
